# Salivary Cytokine Profile as a Possible Predictor of Autism Spectrum Disorder

**DOI:** 10.3390/jcm9103101

**Published:** 2020-09-25

**Authors:** Joanna Samborska-Mazur, Anna Kostiukow, Izabela Miechowicz, Dorota Sikorska, Rafał Rutkowski, Marzena Wyganowska-Świątkowska, Katarzyna Błochowiak

**Affiliations:** 1 Department of Oral Surgery and Periodontology, Poznan University of Medical Sciences, Bukowska 70 Street, 60812 Poznan, Poland; joannasamborska@ump.edu.pl (J.S.-M.); wyganowska@ump.edu.pl (M.W.-Ś.); 2 Department of Rheumatology, Rehabilitation and Internal Diseases, Poznan University of Medical Sciences, 28 Czerwca 1956 135 Street, 61545 Poznan, Poland; annakostiukow@ump.edu.pl (A.K.); dorotasikorska@ump.edu.pl (D.S.); 3 Department of Computer Science and Statistics, Poznan University of Medical Sciences, Rokietnicka 7 Street, 60806 Poznan, Poland; iza@ump.edu.pl; 4 Department of Pathophysiology, Poznan University of Medical Sciences, Rokietnicka 8 Street, 60806 Poznan, Poland; rrutkowski@ump.edu.pl

**Keywords:** autism spectrum disorder, saliva, salivary cytokines, RANTES, neurodevelopmental disorders, autism, eotaxin

## Abstract

Autism spectrum disorder (ASD) is characterized by neurodevelopmental disorders and alterations in immune function and cytokine levels. The aim of this study is to determine the salivary levels of interleukin-1β (IL-1β), interleukin-6 (IL-6), interleukin-8 (IL-8), tumor necrosis factor α (TNFα), monocyte chemoattractant protein-1 (MCP-1), Regulated on Activation, Normal T-cell Expressed and Secreted (RANTES), and Eotaxin in children with ASD and in healthy controlsto assess their predictive potential. We explored correlations between the cytokine levels and the neurodevelopmental disorders related to ASD. The study comprised 19 children with ASD and 19 typically developing (TD) ones. We analyzed salivary levels of IL-1β, IL-6, IL-8, TNFα, MCP-1, RANTES, and eotaxin on Luminex with custom-designed 7-plex kits. The level of RANTES in ASD children was significantly lower than those of TD. In TDs, the salivary levels of IL-1β, MCP-1, and TNFα correlated positively with age. In ASD, the cytokine levels did not correlate with age. There were statistically significant differences between the RANTES level and aggression and gait disturbances, between IL-8 level and fixations/stimulations, and between IL-1β level and no active speech. The levels of the cytokine detected can manifest both systemic and local changes related to ASD. The cytokine pattern cannot be used as a sole ASD predictor, but the salivary levels may be helpful in categorizing the ASD subtype.

## 1. Introduction

Autism spectrum disorder (ASD) is a neurodevelopmental disorder that is characterized by the primary symptoms of impaired social interactions, communication deficits, and repetitive, stereotyped behaviors. These symptoms are caused by genetic, epigenetic, and environmental factors [1,2]. Recent evidence has shown that more than half of the risk for developing ASD can be attributed to genetic mutations and over 40% of the risk is likely to be due to unknown environmental factors [2]. A reliable ASD diagnosis is typically not given until at least 2 years of age, when the disorder starts to manifest [3]. Because of unreliable early diagnosis, effective therapy is usually delayed. Currently, the most effective approach to ASD treatment is social stimulation and behavioral intervention. However, the effectiveness of this therapy depends on initiating treatment as early as possible. The most important strategy is early and reliable diagnosis and conducting a universal screening program for genetic and metabolic disorders to eliminate all potentially harmful environmental factors or detection and the use of reliable ASD predictive biomarkers in the most predisposed children groups [3].

No reliable marker for predicting risk of ASD has been determined so far. There is evidence, however, that a subset of children with ASD presents with alterations in immune function, including differences in immune cell function and numbers, immunoglobulin and cytokine levels relative to typically developing controls [4,5,6]. Furthermore, the immune system plays a significant role in in neurodevelopment and in the regulation of neural plasticity throughout life. There is a direct relationship between the functional status of the immune system and brain function and development [7]. Therefore, the disturbances in cytokine levels in early life can possibly result in neurodevelopmental disorders in later life. Consequently, the altered cytokine levels in ASD may be indicators of a greater deterioration in behavior in future life. It has been suggested that a dysregulation of the immune response may be related to the behavioral and cognitive impairment in ASD. In previous studies, some cytokines were described as promising candidates for predicting the ASD risk. Moreover, the changes in these cytokine levels were correlated with special neurodevelopmental disorders [4,8]. Increased levels of interleukin-6 and -8 (IL-6 and IL-8) were associated with a global ASD phenotype when compared with the general population. An increased IL-8 level was correlated with the presence of intellectual disability, whereas interleukin-12, p70 (IL-12p70), and eotaxin-1 (CCL11) and granulocyte chemotactic protein 2 (GCP2) were associated specifically with the absence of intellectual disability [4]. These correlations between the cytokine levels and the occurrences of special deficits can be a basis for dividing ASD into several subgroups [8]. Furthermore, these cytokines and their connections with special neurodevelopmental disorders may play the role of biomarkers in guiding treatment selection and more individualized therapies. Identifying objective markers of neurodevelopmental disorders related to a subgroup in ASD may assist in reducing the heterogeneity of ASD patients and may lead to the identification of more targeted treatments of ASD-related symptoms.

Cytokines are widely distributed and can be detected in all body fluids. They can be easily determined. In previous studies, mainly serum cytokines levels were used as predictors of ASD [9]. Moreover, the mass spectrophotometry studies revealed changes in selected cytokines in urine, cerebrospinal fluid, and in brain tissue samples in ASD patients [8,9]. We suppose that the salivary cytokine levels could be altered in the ASD group.

We elected to use saliva sampling for the following reasons. Firstly, saliva is rich in cytokines and reflects the concentration of these molecules in serum. Saliva is a functional equivalent to serum in terms of reflecting the health status of the human body. Moreover, salivary proteins are used as biomarkers in common neurological diseases [10]. Secondly, saliva is easily obtainable so the stress associated with the procedure of saliva sampling in ASD patients is reduced. Saliva sampling is safer than blood or cerebrospinal fluid collection by reducing the risk of pathogen transmission from patients suffering from chronic infection. Thirdly, the close relation between saliva secretion and the nervous system could render saliva a useful source of biomarkers that represent various pathological processes in the nervous system and neurodevelopment [11]. According to previous data, saliva seems to be an appropriate body fluid to measure biomarkers important to autism [9,11].

Despite the progress made in recent years in understanding the role of cytokines and neuroinflammation in the pathogenesis of ASD, more data are needed to better elucidate the predictive value of salivary cytokines and the relationship between the salivary levels of selected cytokines and ASD. To address this need, we measured levels of IL-1β, IL-6, IL-8, tumor necrosis factor α (TNFα), monocyte chemoattractant protein-1 (MCP-1), Regulated on Activation, Normal T-cell Expressed and Secreted (RANTES), and Eotaxin in the saliva of children with ASD and in typically developing (TD). The main aim was to determine the predictive potential of these markers. Additionally, we explored possible correlations between the concentrations of cytokines and other clinical and demographic features determined, and the selected neurodevelopmental disorders related to ASD.

## 2. Materials and Methods

### 2.1. Study Groups

A total of 38 children participated in the study. The study comprised 19 children diagnosed with ASD (male:female ratio 18:1, age range 3–13 years) and 19 neurotypical children who were included as typically developing (TDs) (male:female ratio 15:4, age range 3–12 years). The mean age in ASD group was 6.78 years (SD = 2.80 years). The mean age in TD group was 6.84 years (SD = 2.52 years). All children were recruited consecutively in 2018 from schools and kindergartens in the city of Poznan, Poland. A routine patient’s history, including the occurrence of systemic diseases and neurodevelopmental disorders, was taken from teachers and parents. The assessment of developmental disorders occurrence was based on interviews, questionnaires and observations of children conducted among parents, teachers and psychologists. ASD group met the diagnostic criteria of ASD. TD group did not meet the diagnostic criteria of ASD. The diagnostic criteria of ASD followed the 5^th^ edition of Diagnostic of Statistical Manual of Mental Disorders (DSM-5) [12], and were determined by both a child psychiatrist and a developmental psychologist. In the ASD group autism diagnostic observation schedule (ADOS-2) was implemented to confirm the diagnosis [13]. Exclusion criteria included the presence of inflammatory diseases, salivary gland inflammatory diseases, other neuropsychiatric diseases, and pharmacological therapy of ASD. Children did not take any drugs which could have interfered with salivary flow. The protocol for this study was approved by the Bioethics Committee of Poznan University of Medical Sciences, Poland (293/18). This study was performed in accordance with the ethical standards laid down in an appropriate version of the World Medical Association Declaration of Helsinki. Written informed consent was obtained from the parents of each child before any study procedure was carried out.

### 2.2. Dental Examination

Dental examinations were performed in each subject. Two dentists carried out a focused oral exam to assess the number of teeth with caries, the presence of dental plaque, and orthodontic defects. The dental examination was carried out with the use of a mirror and a dental probe under artificial lighting. The state of oral hygiene and the caries prevalence were assessed by Simplified Oral Hygiene Index (OHI-S) and Decayed Missing Filled Tooth (DMFT) index, respectively as described previously [14]. The incidence of caries was determined using dmft index (decayed, missing, filled teeth) which is the sum of teeth with caries (D), teeth extracted because of caries (M), and teeth filled because of caries (F). This parameter was calculated for permanent teeth (DMFT) and milk teeth (dmft).

### 2.3. Sample Collection

Unstimulated mixed saliva was collected from all patients between 9:00 and 11:00 a.m., with a Salivette cotton roll (Sarstedt, Nümbrecht, Germany) as described previously [15]. One hour before sampling, ASD and TD patients refrained from eating, drinking, mouth rinsing, and teeth brushing. Salivette rolls were chewed for 5 min and then placed in special centrifugation tubes. Each sample was refrigerated or stored on ice until arrival at the laboratory, which was no longer than 45 min after the sample had been collected. All the samples were first weighed and then centrifuged at 4500× *g* for 10 min at 4 °C. The supernatant from whole saliva samples obtained by passive flow and saliva samples from the Salivettes were transferred to 1.5 mL Eppendorf tubes and stored at −80 °C for a maximum of three months until analysis. In all cases, the minimum volume of saliva obtained was 1 mL.

### 2.4. Measurement of Salivary IL-1β, IL-6, IL-8, Rantes, Eotaxin, MCP-1, and TNFα

Supernatants were analyzed on Qiagen Liquichip apparatus (Luminex, Austin, TX, USA) with custom-designed 7-plex kits (BioRad, Warsaw, Poland) for the following analytes: Eotaxin, RANTES, IL-6, IL-8, IL-1β, MCP-1, and TNFα using a standardized method as previously described [16]. All saliva supernatants collected were suitable for analysis. Duplicate testing for cytokine determinations for each saliva sample was done to secure testing reliability. The sensitivity and specificity of the tests were assessed using the 5-parameter Receiver Operating Characteristic (ROC) analysis and the Bio-plex Manager software in accordance with the recommended method as described previously [17,18]. ROC curves were generated for each cytokine and then the area under the curve (AUC) and the optimal cut-off values were determined for each parameter using the Bio-Plex Manager software. ROC curves are presented in the supplementary materials.

### 2.5. Statistical Analysis

The calculations were carried out with Microsoft Excel 2016 and STATISTICA software (v.12 StatSoft Inc., Tulsa, OK, USA). Continuous data are presented as medians and interquartile ranges (IQR) or mean and standard deviation (±SD). Distributions of continuous variables were evaluated for normality using the Shapiro-Wilk test. Assessment of the normality distribution of variables is presented in Table 1. Depending on the normality of the distribution of variables, the differences between the two groups were tested using the Mann-Whitney U test or t-test. Categorical variables are presented in contingency tables and their associations were tested, depending on the number of cases, with Fisher’s exact test. Spearman’s rank correlation analysis was used to find the associations between age and the levels of selected cytokines and other clinical parameters, *p* < 0.05 was considered statistically significant.

## 3. Results

All the subjects were of Caucasian origin. Table 2 and Table 3 present a demographic, clinical and laboratory profile of the ASD and TD patients.

### 3.1. Salivary IL-1β, IL-6, IL-8, Rantes, Eotaxin, MCP-1, and TNFα Levels

The salivary RANTES level in ASD children was significantly lower than those of TD (*p* = 0.0314). There were no differences in salivary levels of IL-1β, IL-6, IL-8, eotaxin, MCP-1, TNFα between ASD and TD groups. Detailed results are presented in Table 4.

### 3.2. Correlations Between the Cytokine Levels and Age in TD and ASD groups

In ASD, the salivary levels of IL-1β, IL-6, IL-8, RANTES, eotaxin, MCP-1, and TNFα did not correlate with age (Table 5).

**Figure 1:**
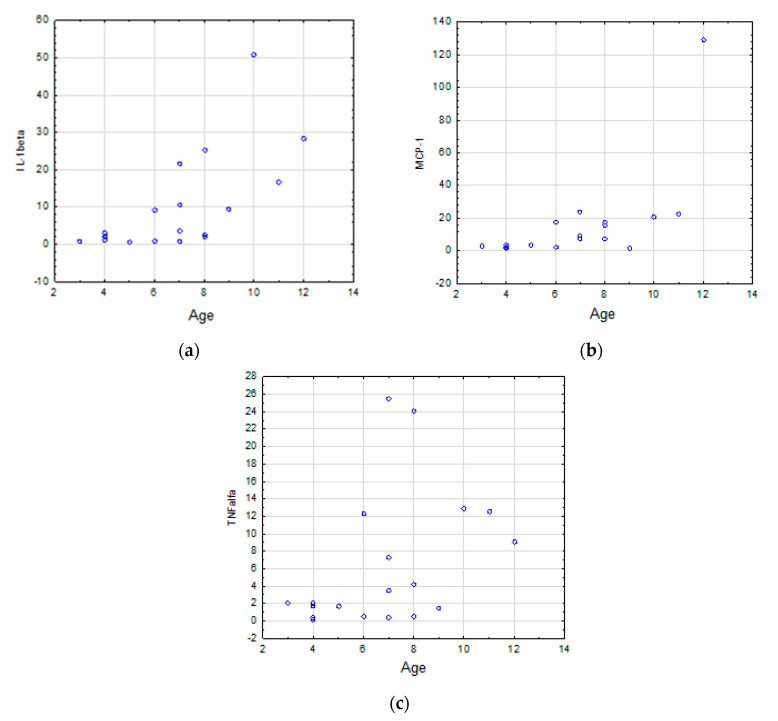
(**a**–**c**) Correlations of salivary levels of IL-1β (Figure 1a), MCP-1 (Figure 1b), and TNFα (Figure 1c) and age in the TD group. The strength of the correlations was determined using Spearman’s rank correlation coefficient; *p* < 0.05 was considered statistically significant.

In TDs, the salivary levels of IL-1β (*r_s_* = 0.70, *p* = 0.0006), MCP-1 (*r_s_* = 0.62, *p* = 0.0039), and TNFα (*r_s_* = 0.4838, *p* = 0.0358) correlated positively with age (Table 6), (Figure 1a–c, respectively).

### 3.3. Correlations between Salivary Levels of Detected Cytokines and Neurodevelopmental Disorders

**Figure 2:**
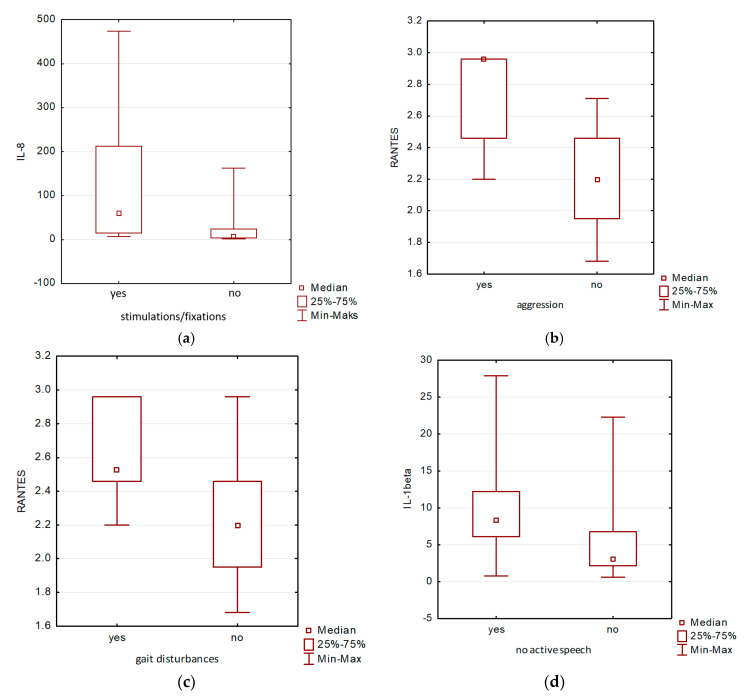
(**a–d**). Statistically significant differences between salivary levels of IL-8 and stimulations/fixations (Figure 2a), levels of RANTES and aggression (Figure 2b) and gait disturbances (Figure 2c) and levels of IL-1β and no active speech (Figure 2d).

There were statistically significant differences between the RANTES salivary level and aggression (*p* = 0.0301), between IL-8 salivary level and fixations/stimulations (*p* = 0.0324), and between gait disturbances and RANTES salivary level (*p* = 0.0447), and between no active speech and IL-1β (*p* = 0.0438), respectively (Figure 2a–d).

## 4. Discussion

Despite the clear relationship between the levels of selected cytokines, and the risk of ASD development and ASD-related neurodevelopmental disorders, the exact nature of these relationships and the predictive potential of salivary cytokines in ASD remain to be fully elucidated. In the present study, we analyzed salivary levels of IL-1β, IL-6, IL-8, RANTES, Eotaxin, MCP-1, and TNFα to determine their potential values as prognostic and predictive markers for ASD. We also assessed the correlation between the salivary levels of these cytokines and ASD-related neurodevelopmental disturbances. Likewise, we were able to find a correlation between age and salivary levels of IL-1β, TNFα, and MCP-1 in TD children. Finally, no significant correlation between age and salivary levels of cytokines in the ASD group was noticed. Taken together, these findings suggest that among salivary cytokines, RANTES is the most sensitive factor and could be considered as a potential prognostic and predictive factor in ASD as well as helpful in categorizing the ASD subtypes. RANTES was the most sensitive parameter related to the groups that were determined and to accompanying ASD neurodevelopmental disorders.

The only cytokine that presented a significantly lower salivary level in children with ASD compared to TD was RANTES. Previous studies have reported divergent and conflicting results in the plasma levels of the cytokines in the ASD group compared to controls [7,8]. The conflicting results in selected cytokine levels presented in our study, and in other studies, too may result from ASD heterogeneity. Among ASD phenotypes various abnormal adaptive responses have been reported including increased T lymphocyte cell production, overexpression of Th1-like cytokines, and a skewing of cytokine responses toward a Th2 cytokine profile [19]. Furthermore, a skewing of cytokine response toward Th1 or Th2 cytokines have been reflected by various neurodevelopmental features related to ASD. Overexpression of pro-inflammatory cytokines and Th1 skewing have been associated with more impaired behaviors whereas Th2 response have been associated with improved developmental and adaptive functions in ASD [19]. Increased production of Th2 cytokines can be beneficial in modulating and improving behaviors in some individuals with ASD. Although our results revealed neither Th1-like cytokine nor Th2-like cytokine predominance; however, decreased RANTES level can indicate the Th1/Th2 activity dysregulation. Moreover, real Th1 and Th2 activity could be masked by the presence of different ASD phenotypes in our study group. RANTES, detected in our study, belongs to the chemokine family, stimulating recruitment of well-defined leukocyte subsets. It is involved in homeostasis between Th1 and Th2 activity. Its decreased level can reflect an imbalance in their proportions. Masi et al. revealed increased RANTES plasma levels with concurrent lower proportions of Th1 cells as well as higher proportions of Th2 cells in children with ASD compared to TD, providing evidence of an imbalance of Th1-and Th2-like cytokines in ASD [8]. Moreover, peripheral blood mononuclear cells (PBMC) in the ASD group showed increased activation of both the Th1 and Th2 arms of the immune response and a Th2 dominance without a compensatory increase in the expression of other regulatory cytokines [8,20]. The decreased salivary level of RANTES in our study did not correlate with the changes in other cytokine levels. This could result from a possible imbalance in both Th-1 and Th-2-like cytokine profile in saliva and, additionally, the non-existence of possible compensatory mechanisms reflected in other cytokine levels. Similar results to those presented in our study were obtained by Manzardo et al. who reported decreased plasma cytokine levels representing chemokines involved in the T-helper cell immune system and hematopoiesis in the children with ASD compared with unrelated siblings without ASD [21]. Similar data were also detected by Gładysz et al. who reported decreased RANTES levels in newborn peripheral blood obtained from archive bloodspots within 24–48 h after birth [22]. The possible explanation for the decreased plasma levels of these selected cytokines is increased cellular activity in the ASD group. The intensity of immune cell activation can result in the increased consumption of the detected cytokines [22]. Other well-known alternation in the cytokine profile in ASD is an overexpression of pro-inflammatory cytokines. Increased levels of selected pro-inflammatory cytokines produced by PBMC have been shown to disrupt neurodevelopment in ASD groups [23,24]. These pro-inflammatory cytokines can modulate responses in the central nervous system and can alter neurodevelopment. An aberrant immune response during neurodevelopment could lead to changes in early brain development and produce a neurological dysfunction related to ASD [24,25,26,27,28]. Some previous studies reported elevated levels of pro-inflammatory cytokines such as TNFα and IL-1β in children with ASD compared to typically developing children [21,23]. According to a meta-analysis obtained by Saghazadeh et al. providing data on circulating concentrations of pro-inflammatory cytokines, in people with ASD compared to the controls there were medium increases in the levels of serum IL-1β and small increases in the levels of blood IL-1β and both serum IL-6 and TNFα for patients with ASD [7]. As in our study, Ashwoodd et al. reported decreased RANTES level in the ASD group compared to the controls [24]. Zafarullah et al. revealed decreased levels of the chemokines Eotaxin, MCP-1, and RANTES in both the Fragile X syndrome with autism spectrum disorder (FXS + ASD) and Fragile X syndrome without autism (FXS) groups compared to the controls. Moreover, the levels of Eotaxin, MCP-1, and RANTES were significantly different for FXS + ASD compared to FXS. There was a significantly distinct profile of the cytokines: Eotaxin, RANTES, and MPC-1 in FXS + ASD compared to the controls [28]. It is unknown whether the cytokine changes are determinants in the development of FXS and if they occur throughout the lifetime of FXS [28]. Usually the cytokines belonging to the same either pro-inflammatory or anti-inflammatory groups showed similar results [23]. However, overlapping functions of these cytokines were not reflected in their similar levels in saliva in the ASD group in our study. The relatively small study groups may have affected the lack of differences in the expression of other cytokines. These findings also support the evidence that pro-inflammatory cytokine levels can show marked differences within ASD subgroups.

Apart from the differentially induced Th1 and Th2 cells in ASD, other key player in the pathogenesis of some autoimmune neuroinflammatory disease traditionally attributed to Th1 cells is Th17. IL-17A is a pro-inflammatory cytokine that is produced by a number of human immune cells, including Th17 cells, neutrophils, and PBMC. Children with ASD have significantly higher serum IL-17A levels than typically developing children. Moreover, patients with severe ASD have significantly higher serum IL-17A levels than children with mild to moderate ASD. IL-17 can regulate the induction and generation of Th1 responses via induction of IL-12 and IFN-γ in antigen-presenting cells [29]. Although IL-17 have not been detected in our study, IL-17 seems to be an important modifier of Th1-like cytokine levels in some ASD subgroups.

The expression of cytokines that was also detected in our study produced divergent results depending on the type of body fluid or body tissue. The levels of the same cytokine were differentially detected in serum, plasma, PBMC, cerebrospinal fluid, amniotic fluid, and in the brain [26,27]. It seems that a universal and most reliable body fluid or body tissue should be found for assessing the cytokine profile in the ASD group. For understanding the role of decreased salivary levels of RANTES in ASD, its levels in other body fluids need to be comprehensively established. In our opinion, salivary cytokine levels should be always compared to other body fluids in the same study groups. Although many studies have reported altered levels of cytokines or abnormal immune function in ASD, it has been difficult to identify a consistent pattern of immune alterations across the various studies or explain this change. The discrepancies among the studies may be due to the subjects’ gender, ASD subgroups, the analytical techniques and the tissue specimen types.

Cytokines such as RANTES can have an endocrine, paracrine, and autocrine function depending on the distance between the cell secreting the signaling ligand and the cell receiving the chemical signal. The changes in RANTES levels in our study can be reflected in saliva via an autocrine pathway. The levels of cytokines in saliva do not correspond with their serum levels reported previously [24]. One of the possible explanations of this finding is the change in the microbiological environment of the oral cavity. Salivary cytokine changes in ASD can reflect local immune responses to the microbiological changes in the oral cavity. There is a significant difference in bacterial diversity in the oral cavity between ASD and the control [30]. Moreover, transcriptional activity of the salivary and dental microbiota in ASD patients differs from that of TD children [30]. ASD is associated with abnormalities such as buccal sensory sensitivity, taste and texture aversions. Therefore, oral hygiene and dental treatment are difficult to perform in children with ASD. ASD children are characterized by a higher prevalence of caries, poor oral hygiene, and extensive needs for dental treatment compared to children without autism. Qiao et al. observed worse oral health status among children with ASD [31,32]. According to Bartolome-Villar et al. a greater percentage of ASD children needed dental treatment, compared to 40% in the control group [33]. Moreover, some studies have revealed the greater prevalence of gingivitis, periodontitis, and gingival bleeding [33]. Similar results were obtained in our study, where children with ASD were characterized by a lower ratio of constant contact with a dentist, worse oral hygiene, and higher caries prevalence. This could increase the amount of selected bacterial species and change the bacterial flora. Some of the features related to ASD, such as restricted diet, repetitive hand-to-mouth behaviors, resistance to oral hygiene, and genetic polymorphisms associated with immune function, can lead to oral dysbiosis and immune response to the microbiological changes in the oral cavity. Qiao et al. reported damaging oral habits in ASD children group, including mouth breathing, self-injurious habits, object biting, and significantly higher rates of halitosis and oral pain and lesions, which were linked to toothache, oral ulceration, and dental and mucosal traumatisms [31,32]. All these conditions trigger the immune reactions that can be reflected in salivary cytokine levels. Another source of the changes in the salivary cytokine profile in ASD children is the spontaneous production of pro-inflammatory cytokines by mucosal lymphocytes, as well as the production of cytokines against common dietary proteins in ASD patients who additionally manifest gastrointestinal symptoms [34,35]. Moreover, some ASD patients presenting with gastrointestinal complaints and non-IgE-mediated food allergy manifest changes in monocyte activity and a monocyte-dependent, pro-inflammatory cytokine profile [22,36]. Finally, cytokines in saliva may also be of a nonglandular origin. The saliva samples used in our study should be treated as a mixture of the salivary glands and other fluids that originate from the oropharyngeal mucosa, oral mucosal transudate, fungi, bacteria, viruses, and gastrointestinal reflux liquid [37]. Taken together, these local changes and conditions in the oral cavity environment can be reflected by the changes in salivary cytokines.

In our study, in the TD group, the salivary levels of MCP-1, TNFα, and IL-1β levels correlated with age, contrary to the ASD group. The independence of age increases the importance of the level of selected cytokines in the early diagnosis of ASD as typically developing children do show a relationship of the level of selected cytokines with age. The changes in cytokine profile related to ASD may occur in the early stages of life and determine the future neurodevelopment. Similar results were obtained by Decker et al. who reported a decrease of IL-1ra and TNF-α levels with age in TD children. This decrease was calculated to be between 9% and 4% per year of age [38]. IL-6 and TNF-α levels were higher in children aged 7–17 years compared to adults and younger children [39]. The same meta-analysis reported that IL-1β level was not influenced by age [39]. These observations are contrary to our results. TNFα seems to be strongly correlated with age in TD children. Moreover, TNFα level is prone to physiological changes that occur during adolescence [40]. According to Kleiner et al. IL-8 and RANTES levels are constant throughout the development in TD similar to our results. However, MCP-1 levels showed differences with age in contrast to our results [40]. Changes in cytokine levels with age in TD subjects may reflect the degree of immune system development and the maturity of the immune response. Moreover, developmental differences between girls and boys may be reflected in the correlation between age and the cytokine levels. Masi et al. reported that an overall decline in cytokine levels was associated with aging in ASD males only [41]. The physiological changes occurring during the onset of puberty and beyond may contribute to the variations of cytokine levels with age and sex. For a comprehensive assessment of age impact on the cytokine levels in both ASD and TD children, a longitudinal analysis throughout child development in the same gender study group seems to be a valuable method to evaluate the cytokine levels diagnostic and predictive significance in ASD. Moreover, this dynamic research model could let correlate salivary cytokine levels with neurodevelopmental disorders related to ASD. A relatively small group of ASD children of similar age in our research makes the correlation between age and the cytokine levels less reliable. 

The changes in cytokine profile and the predominance of pro-inflammatory cytokines in the early stage of neurodevelopment may play a role in the pathophysiology of ASD-related disorders. Elevated inflammatory mediators may be associated with abnormal behaviors in individuals with ASD. They have been linked to a deterioration in communication skills, stereotyped behaviors and hyperactivity, and impairment of cognitive functions [42,43]. Some previous studies highlighted associations between increased pro-inflammatory cytokine levels and behavioral impairments in ASD as well as between peripheral immune system abnormalities in ASD and pro-inflammatory bias [44,45]. Higher expression of IL-1β, IL-6, and IL-8 were correlated with more impaired stereotyped patterns of behavior while increased MCP-1, RANTES, and eotaxin levels were associated with worsening behavioral symptoms and cognitive and adaptive ability [44,45]. Some of these previous findings were consistent with the results presented in our study. On the other hand, the other studies reported that increased IL-8 level was correlated with higher severity of social impairments in females only [43]. DNA analysis suggests that females could be more resilient to genetic insults due to carrying more extreme neurodevelopmental-related genetic mutations than males with the same symptoms. These findings emphasize that gender to play an important moderating role in severity of neurodevelopmental disorders related to ASD. In our study, we detected several correlations between the salivary levels of selected cytokines and selected neurodevelopmental disorders. Increased levels of these cytokines correlated with the prevalence of neurodevelopmental abnormalities. Some of the disorders detected in our study and dependent on cytokine levels are responsible for hyperactivity related to ASD. Han et al. noted higher RANTES levels in ASD children with attention deficit hyperactivity disorder (ADHD) compared to ASD without ADHD [46]. Self-harm, simulations/fixations and gait disturbances can manifest concentration deficits and symptoms of hyperactivity. Enstrom et al. reported that elevated IL-1β had been correlated with poor verbal contact in the ASD group [47], which was similar to our results. Moreover, IL-1β seems to be an important marker for deficits in communication and language and for the development of communication skills in ASD. Strong correlations between elevated RANTES levels and the development of motor skills and deficits in this area in ASD were described previously in some studies [19,48]. Elevated levels of pro-inflammatory cytokines also increase the risk of intellectual disability and several other neurodevelopmental disorders, suggesting that these cytokine alternations generally increase the vulnerability of the developing brain to developmental defects. Moreover, cytokine levels in the blood in the ASD group reflect changes in cytokine levels in the brain. Increased levels of IL-1β, IL-6, IL-8 were specifically associated with a regressive form of ASD and more impaired stereotypical behaviors [43]. Moreover, Estes et al. reported that mutations in genes for several members of the IL-1 cytokine receptor family were associated with ASD and ASD-related disorders [43]. Furthermore, this mutation was originally identified for genes related to X-linked intellectual disability [43]. According to Heuer at al., the increased levels of IL-6 and IL-8 were associated with a global ASD phenotype when compared with TD, but only IL-8 was correlated with both the presence and the absence of intellectual disability. In the same study, the increased IL-8 was associated with the early onset of autism, while the increased IL-10 and eotaxin levels were correlated with the regressive ASD subtype [5]. In contrast to this study, Gomez-Fernandez et al. reported no differences in IL-1β, IL-6, IL-8, MCP-1, and RANTES plasma levels between ASD children with neurodevelopmental regression and without neurodevelopmental regression [20]. Previous studies reported a close relationship between cytokine imbalance and changes in cytokine levels and several processes regulating brain development, including neurogenesis, synapse formation, and plasticity, from early prenatal CNS development to postnatal development and adulthood [37]. Divergent results may originate from the different time of neurodevelopment and the different period of cytokine influence on the brain development. It is important to consider whether the limitation of the neuroinflammation period could be an adaptive mechanism to allow the brain to cope with ASD-related deficits [43].

The limitations of the study result from the lack of familial and maternal history. Maternal infections can lead to the release of pro-inflammatory cytokines and activation of Th17 cells in the mother’s bloodstream. This acute immune activation caused by viral infection during the first trimester of pregnancy may determine the immune status of the fetus and its vulnerability to ASD [4,8,49]. Furthermore, familial autoimmunity implicated in a maternal history of rheumatoid arthritis, celiac disease and type 1 diabetes can increase the risk of autism [8]. Another limitation of the study is the heterogeneity of the ASD group related to the children’s neurodevelopmental profile and gender. Sex is believed to play an important role in the development. ASD manifestations differ in both female and male groups. Females present with different characteristic phenotypes in ASD. Moreover, gender is postulated to be a potential modifier of the relationship between altered cytokine profile and the severity of ASD-related symptoms. Therefore, differences in gender distribution between ASD and TD groups can interfere with our results. Furthermore, a relatively small study group did not allow the elimination of the potential influence of behavioral and neurodevelopmental gender specific differences among ASD children on the salivary cytokine levels determined in our study. Presence of different neurodevelopmental ASD subtypes disturbs the assessment of correlations between the salivary cytokine levels and selected neurodevelopmental disorders. More reliable data could result from the possible relationship between cytokine aberrations and the behavioral impairments occurring in the selected ASD subtypes. These limitations are the main handicaps in identifying potential biomarkers in ASD diagnosis. Therefore, our results should be verified in a wider and more homogenous group of patients. A comprehensive study researching possible correlations between ASD and changes in cytokine profile requires the determination of cytokine levels in other body fluids, especially in blood serum, and correlations of their levels both in serum and in saliva. In our opinion, the salivary levels of detected cytokines can be triggered by local immune and microbiologic changes determined by special habits and predispositions related to ASD. For the identification of the potential salivary biomarkers in ASD, the comprehensive assessment of the same cytokines in both saliva and serum is required in order to exclude all possible local impacts and conditions. Furthermore, all of the dental examinations were performed by the same dentists in our study without the inter-rater agreements between the examiner and another experienced dentist. A lack of the reliability test in the dental examination may have interfered with the correlations between salivary cytokine levels and the dental parameters. Although ASDs do not disturb salivary flow and do not trigger any changes in salivary glands morphology the possible changes in salivation could have induced qualitative alternations in the salivary samples. Theses possible correlations were observed in other neurodegenerative diseases such as Alzheimer’s disease, dementia, and Parkinson’s disease [50,51,52]. Therefore, the comprehensive quantitative and qualitative salivary analysis is recommended to exclude the influence of decreased salivation on the level of saliva markers detected in our study.

## 5. Conclusions

The salivary cytokine profile in ASD children differs from TD but may reflect the local immune conditions triggered by microbiological environmental changes in the oral cavity in the ASD group, as well as immune abnormalities related to ASD. The salivary levels of the cytokine detected can manifest both systemic and local changes related to ASD. 

Confirmation of salivary changes in the cytokine profile in blood samples allows the potential influence of local changes in oral cavity for cytokine alternations in ASD and related neurodevelopmental disorders to be excluded. It seems that correlations between selected neurodevelopmental disorders and levels of salivary cytokines have a more important value in early diagnosis and in effective treatment. Furthermore, cytokine profiles in children who had already been diagnosed with ASD could be used as follow-up biomarkers to monitor the progression of ASD after behavioral intervention. The association between immune system dysfunction and behavioral abnormalities, in at least a subset of individuals with ASD, suggests a potential role for immunomodulatory therapies as a causative treatment. Because of the presence of the heterogeneity of neurodevelopmental and immune disorders related to ASD and the heterogeneity of ASD, it seems that, in general, the cytokine pattern cannot be used as a sole and reliable ASD predictor, but the salivary levels of the cytokines reflecting the alternations of immune conditions may be helpful in categorizing the ASD subtype.

## Figures and Tables

**Table 1:** Assessment of the normality distribution of variables.

Cytokine	Shapiro-Wilk Test *p*-Value	
	ASD (*n* = 19)	TD (*n* = 19)
IL-1β	**0.0149**	**0.0002**
IL-6	**0.0001**	**<0.0001**
IL-8	**0.0009**	**<0.0001**
Eotaxin	**<0.0001**	0.0838
MCP-1	0.0677	**<0.0001**
RANTES	0.3889	0.5419
TNFα	**0.0008**	**0.0005**

**Table 2:** Detailed characteristics of laboratory, demographic and clinical parameters in ASD and TD groups.

Clinical, Demographic and Laboratory Data	Autism Spectrum Disorder (ASD)	Typically Developing (TDs)	*p*
Number of individuals, *n*	19	19	
Age (y) mean (±SD)	6.78 (±2.80)	6.84 (±2.52)	0.7123 ^a^
Gender, *n* (Female/Male)	1/18	4/15	0.3397 ^b^
Vision disturbances, *n* yes/no	12/7	0/4	**0.0023 ^c^**
Self-injury, *n* yes/no	4/15	1/3	0.2253 ^c^
Aggression,			**0.0323 ^c^**
^1^*n* yes/rather yes	0/2	2/0	
^1^ no/rather no	13/2	2/0	
Fixations simulations,			**0.0016 ^c^**
*n* yes/rather yes	13/3	0/0	
*n* no/rather no	1/2	4/0	
Gait disturbance,			<1.0000 ^c^
*n* yes/rather yes	2/3	1/0	
*n* no/rather no	12/2	3/0	
Echolalia,			0.1246 ^c^
*n* yes/rather yes	9/1	0/0	
*n* no/rather no	6/3	4/0	
Sleeping disturbance,			0.7431 ^c^
*n* yes/rather yes	2/1	0/0	
*n* no/rather no	11/5	4/0	
Defecation disturbance,			0.5138 ^c^
*n* yes/rather yes	1/1	0/0	
*n* no/rather no	11/6	4/0	
Food selectivity,			0.7976 ^c^
*n* yes/rather yes	6/4	2/0	
*n* no/rather no	6/3	2/0	
Auditory hypersensitivity,			0.1286 ^c^
*n* yes/rather yes	8/4	1/0	
*n* no/rather no	3/4	3/0	
Olfactory hypersensitivity,			0.7945 ^c^
*n* yes/rather yes	2/1	1/0	
*n* no/rather no	12/4	3/0	
No active speech,			0.2298 ^c^
*n* yes/rather yes	8/2	0/0	
*n* no/rather no	7/2	4/0	
Tantrums of hysteria, crying,			0.4814 ^c^
*n* yes/rather yes	6/4	2/0	
*n* no/rather no	4/5	2/0	
Repetitive, stereotyped behaviors,			0.6224 ^c^
*n* yes/rather yes	4/4	0/0	
*n* no/rather no	8/3	3/1	

SD, standard deviation, *n*, number, ^1^ the distribution of developmental disorders presented in the table was made on the basis of answers given in questionnaires or interviews by parents, teachers and psychologists as “yes,” “rather yes,” “no,” “rather no,” ^a^ Mann-Whitney U test, ^b^ Fisher’s exact test, ^c^ Fisher-Freeman-Halton’s test.

**Table 3:** Detailed characteristics of clinical parameters from the dental examination in Autism spectrum disorder (ASD) and typically developing (TD) groups.

Dental Data.	Autism Spectrum Disorder (ASD)	*p*	Typically Developing (TDs)
Previous contact with a dentist, *n* yes/no	9/10	0.0911 ^a^	15/4
Constant contact with a dentist, *n* yes/no	2/17	**0.0018 ^a^**	12/7
Number of teeth with caries, *n* yes/no,	12/7		5/14
mean (±SD)	2.73 (±3.19)	0.0505 ^b^	1.42 (±3.04)
Oral hygiene, *n* good/bad	7/12	0.5148 ^a^	10/9
Presence of oral plaque, *n* yes/no	6/13	1.0000 ^a^	7/12
Presence of orthodontic defect, *n* yes/no	2/17	0.6598 ^a^	4/15
Presence of caries, *n* yes/no	12/7	**0.0488 ^a^**	5/14
Simplified Oral Hygiene Index (OHI-S)			
mean (±SD)	0.73 (±1.15)	0.9312 ^b^	0.52 (±0.88)
median (a 25–75% confidence interval)	0.00 (0.00–1.83)		0.00 (0.00–0.83)
Decayed Missing Filled Tooth (DMFT) index,			
mean (±SD)	Dmft ^c^ 2.64 (±3.01)	0.1975 ^b^	Dmft ^c^ 2.00 (±3.59)
median (a 25%-75% confidence interval)	DMFTdmft ^d^ 2.89 (±3.22)	0.1298 ^b^	DMFTdmft ^d^ 2.00 (±3.59)
	Dmft ^c^ 2.00 (0.00–4.00)		Dmft ^c^ 0.00 (0.00–3.00)
	DMFTdmft ^d^ 2.00 (0.00–5.00)		DMFTdmft ^d^ 0.00 (0.00–3.00)

^a^ Fisher’s exact test, ^b^ Mann-Whitney U test, SD, standard deviation, *n*, number, ^c^ primary dentition, ^d^ mixed or/and secondary dentition.

**Table 4:** Comparison of IL-1β, IL-6, IL-8, RANTES, eotaxin, MCP-1, and TNFα salivary levels between the ASD and TD groups.

Cytokines	ASD (*n* = 19) Median (a 25–75% Confidence Interval)	TDs (*n* = 19) Median (a 25–75% Confidence Interval)	*p*
IL-1β (pg/mL)	6.84 (3.84–12.2)	2.98 (1.04–16.56)	0.3972 ^b^
IL-6 (pg/mL)	0.67 (0.25–2.16)	0.44 (0.25–2.11)	0.5885 ^b^
IL-8 (pg/mL)	55.36 (15.02–162.75)	47.59 (7.29–117.09)	0.6300 ^b^
Eotaxin (pg/mL)	0.11 (0.08–0.15)	0.08. (0.05–0.16)	0.3248 ^b^
RANTES (µg/mL)	2.46 (2.20–2.59)	2.46 (2.46–2.84)	**0.0314 ^a^**
MCP-1 (pg/mL)	5.43 (3.77–10.39)	7.19 (3.00–17.54)	0.5592 ^b^
TNFα (pg/mL)	6.61 (2.06–21.48)	2.06 (0.54–12.24)	0.1018 ^b^

^a^*t*-test, ^b^ Mann-Whitney *U* test.

**Table 5:** Correlations between IL-1β, IL-6, IL-8, RANTES, eotaxin, MCP-1, and TNFα salivary levels and age in ASD group. The strength of the correlations was determined using Spearman’s rank correlation coefficient; *p* < 0.05 was considered statistically significant.

**Age &**		***n***	***r_s_***	***p***
	IL-1β	19	−0.03	0.899
	IL-6	19	–0.14	0.563
	IL-8	19	–0.18	0.471
	Eotaxin	19	–0.16	0.523
	MCP-1	19	–0.05	0.853
	RANTES	19	0.05	0.840
	TNFα	19	0.15	0.545

**Table 6:** Correlations between IL-1β, IL-6, IL-8, eotaxin, MCP-1, and TNFα salivary levels and the age in the TDs group. The strength of the correlations was determined using Spearman’s rank correlation coefficient; *p* < 0.05 was considered statistically significant.

**Age &**		***n***	***r_s_***	***p***
	IL-1β	19	0.71	**<0.001**
	IL-6	19	0.19	0.438
	IL-8	19	0.40	0.089
	Eotaxin	19	0.42	0.071
	MCP-1	19	0.63	**0.004**
	RANTES	19	0.27	0.271
	TNFα	19	0.48	**0.036**

## Supplementary Materials

The following are available online at https://www.mdpi.com/2077-0383/9/10/3101/s1, Figure S1: Receiver Operating Characteristic (ROC) curves of IL-1β, Figure S2: Receiver Operating Characteristic (ROC) curves of IL-8, Figure S3: Receiver Operating Characteristic (ROC) curves of MCP-1, Figure S4: Receiver Operating Characteristic (ROC) curves of TNFα, Figure S5: Receiver Operating Characteristic (ROC) curves of IL-6, Figure S6: Receiver Operating Characteristic (ROC) curves of Eotaxin, Figure S7: Receiver Operating Characteristic (ROC) curves of RANTES.
