# Supplementary material for: Salivary Cytokine Profile as a Possible Predictor of Autism Spectrum Disorder
Source: J Clin Med. 2020 Sep 25;9(10):3101. doi: 10.3390/jcm9103101 (PMC7601503; doi:10.3390/jcm9103101)
Supplement: Supplementary file 1 [file jcm-09-03101-s001.pdf]

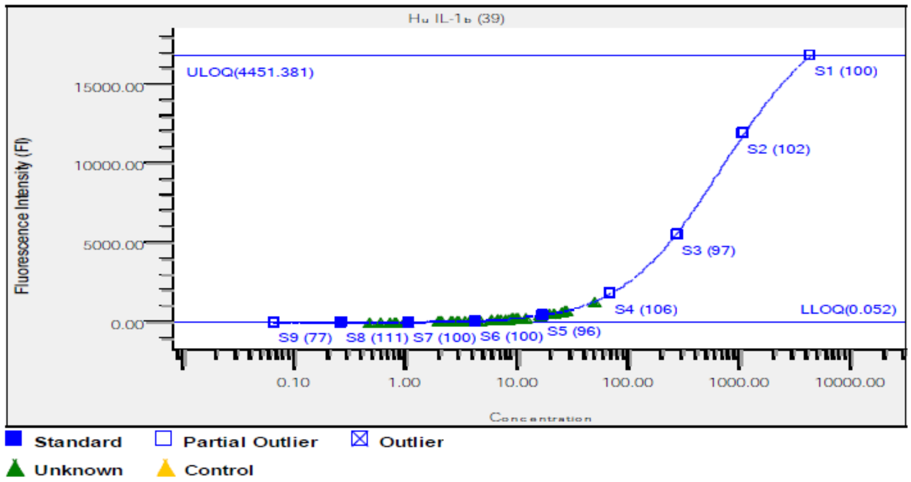


Regression Type: Logistic - 5PL

Std. Curve: FI = 1.24678 + (20200.6 - 1.24678) / ((1 + (Conc / 522.079)^-0.873146))^1.2685

FitProb. = 0.7045, ResVar. = 0.5425

Figure S1. Receiver Operating Characteristic (ROC) curves of IL-1β

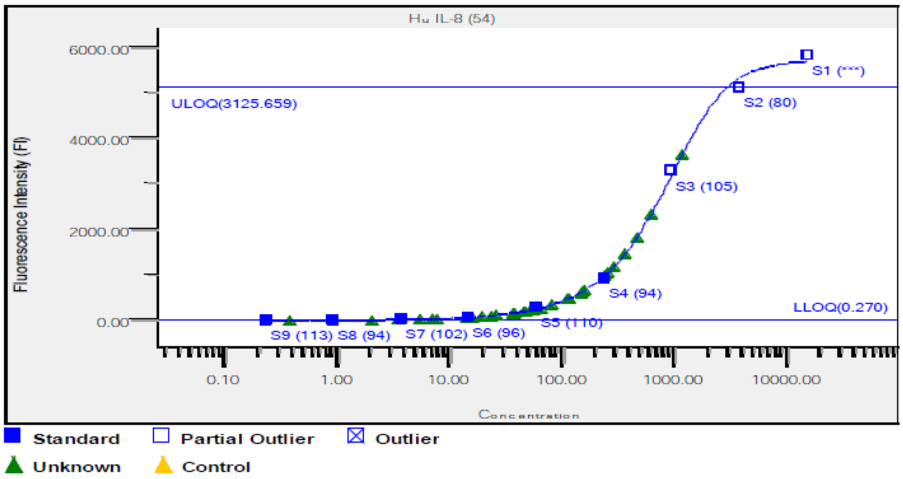


Regression Type: Logistic - 5PL

Std. Curve: FI = 0.677121 + (5726.47 - 0.677121) / ((1 + (Conc / 1396.73)^-1.87252))^0.535948

FitProb. = 0.2138, ResVar. = 1.4524

Figure S2. Receiver Operating Characteristic (ROC) curves of IL-8

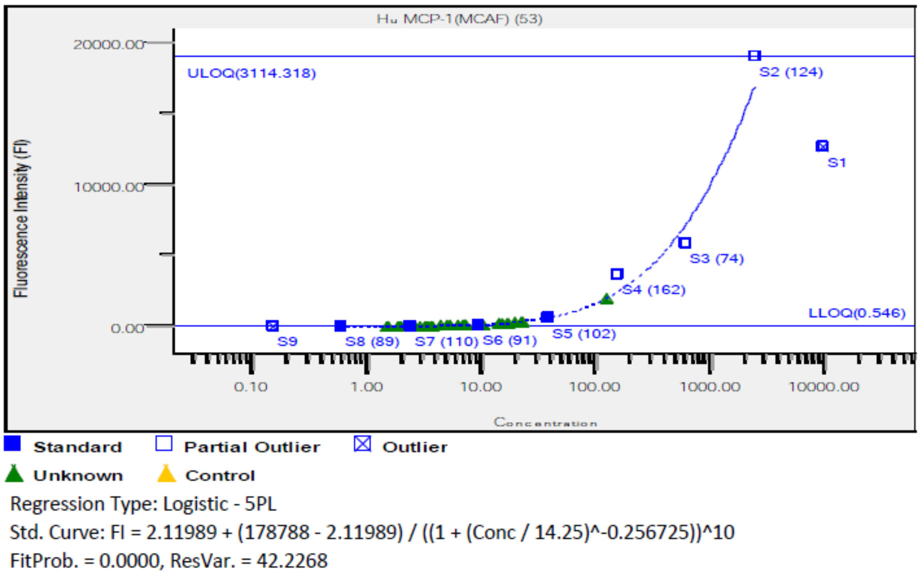


Figure S3. Receiver Operating Characteristic (ROC) curves of MCP-1

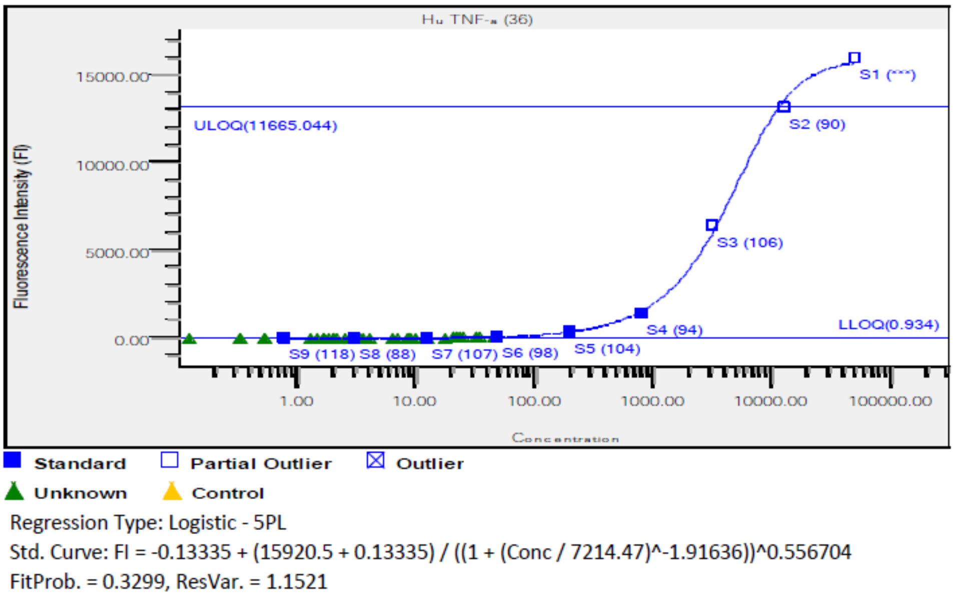


Figure S4. Receiver Operating Characteristic (ROC) curves of TNF$\alpha$

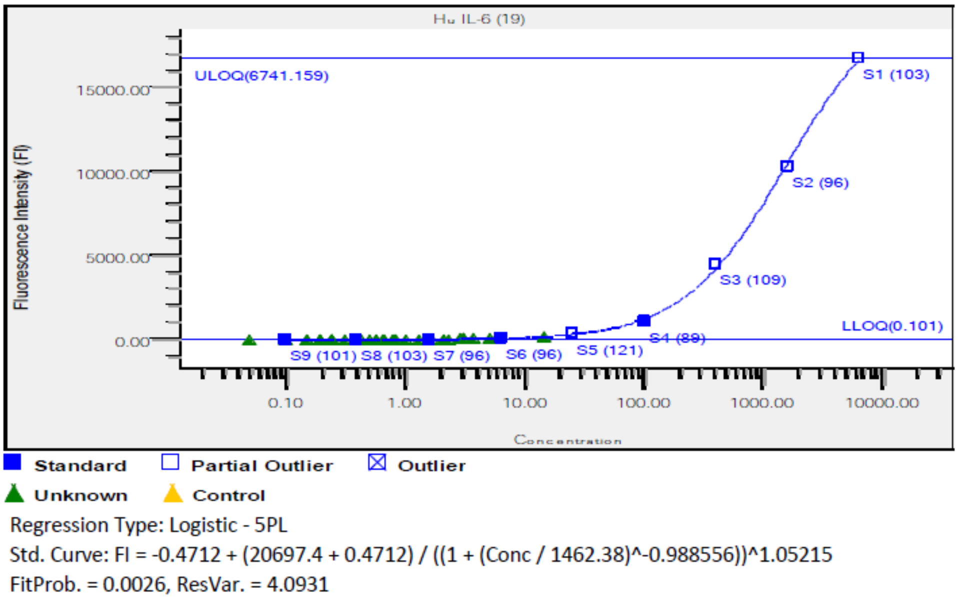


Figure S5. Receiver Operating Characteristic (ROC) curves of IL-6

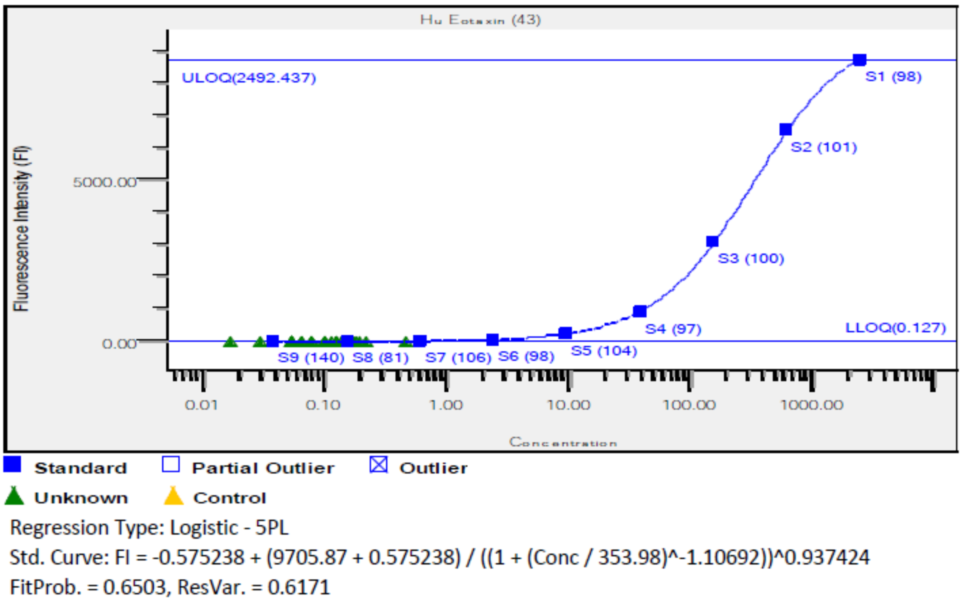


Figure S6. Receiver Operating Characteristic (ROC) curves of Eotaxin

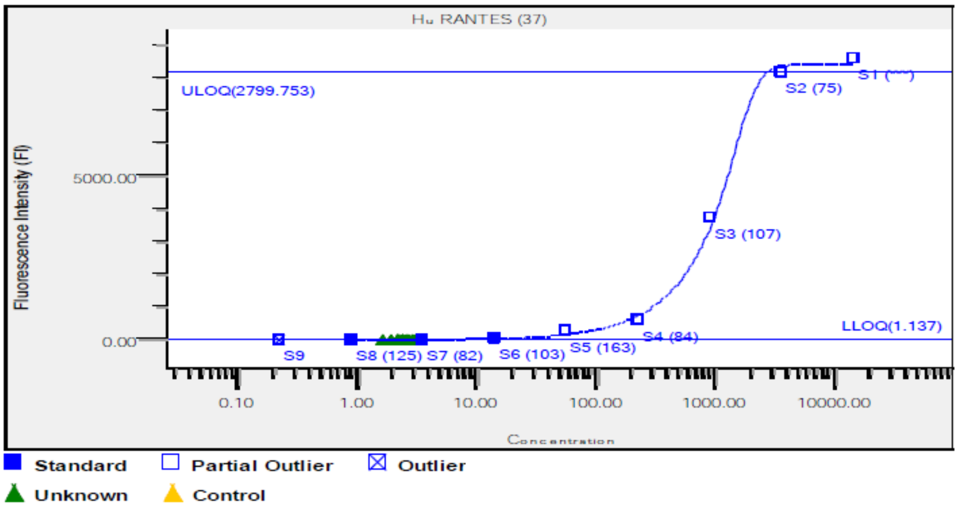


Regression Type: Logistic - 5PL

Std. Curve: FI = -3.05151 + (8434.84 + 3.05151) / ((1 + (Conc / 2051.01)^-5.97239))^0.188739

FitProb. = 0.0000, ResVar. = 24.9551

Figure S7. Receiver Operating Characteristic (ROC) curves of RANTES
